# Supplementary material for: Effectiveness and Safety of Ayurvedic Medicines in Type 2 Diabetes Mellitus Management: A Systematic Review and Meta-Analysis
Source: Front Pharmacol. 2022 Jun 8;13:821810. doi: 10.3389/fphar.2022.821810 (PMC9213670; doi:10.3389/fphar.2022.821810)
Supplement: Supplementary file 1 [file DataSheet1.zip › Supplementary Material/Forest and Funnel Plots/Trigonella foenum-graecum/Subgroup analyses- comparator/Trigonella foenum-graecum_HbA1c_comparator subgroup analysis.pdf]

Trigonella foenum-graecum - HbA1c - comparator subgroup analysis

Study	Intervention N	Intervention Mean	Intervention SD	Comparator N	Comparator Mean	Comparator SD	Mean Diff. with 95% CI	Weight (%)
**No medicine / no additional medicine**								
Ansari 2011	97	8.40	1.90	92	8.68	1.60	-0.28 [-0.78, 0.22]	11.32
Suchitra 2015	30	7.71	3.91	30	7.80	0.57	-0.09 [-1.50, 1.32]	2.86
Kaur 2016	30	7.54	3.91	30	7.46	0.57	0.08 [-1.33, 1.49]	2.86
Singh 2016	20	6.96	0.84	20	7.28	1.06	-0.32 [-0.91, 0.27]	9.69
Ranade 2017	30	6.94	2.94	30	7.40	2.69	-0.46 [-1.89, 0.97]	2.81
Hota 2019	102	5.16	3.55	102	5.65	3.45	-0.49 [-1.45, 0.47]	5.29
Heterogeneity: $\tau^2 = 0.00$, $I^2 = 0.00\%$, $H^2 = 1.00$							-0.30 [-0.62, 0.03]	
Test of $\theta_i = \theta_j$: Q(5) = 0.57, p = 0.99								
**Placebo**								
Gupta 2001	12	7.54	0.90	13	8.14	1.10	-0.60 [-1.39, 0.19]	6.92
Lu 2008	46	6.56	0.95	23	8.20	1.73	-1.64 [-2.27, -1.01]	9.08
Rafraf 2014	44	7.74	1.65	44	8.20	1.95	-0.46 [-1.21, 0.29]	7.36
Kandhare 2018	58	7.25	1.24	53	7.74	1.30	-0.49 [-0.96, -0.02]	11.91
Hassani 2019	62	7.44	1.27	63	7.96	1.29	-0.52 [-0.97, -0.07]	12.39
Rashid 2019	32	6.23	0.58	32	7.18	0.17	-0.95 [-1.16, -0.74]	17.52
Heterogeneity: $\tau^2 = 0.10$, $I^2 = 64.23\%$, $H^2 = 2.80$							-0.79 [-1.12, -0.45]	
Test of $\theta_i = \theta_j$: Q(5) = 12.61, p = 0.03								
**Overall**							-0.62 [-0.87, -0.36]	
Heterogeneity: $\tau^2 = 0.09$, $I^2 = 51.61\%$, $H^2 = 2.07$								
Test of $\theta_i = \theta_j$: Q(11) = 21.99, p = 0.02								
Test of group differences: $Q_b(1) = 4.22$, p = 0.04								

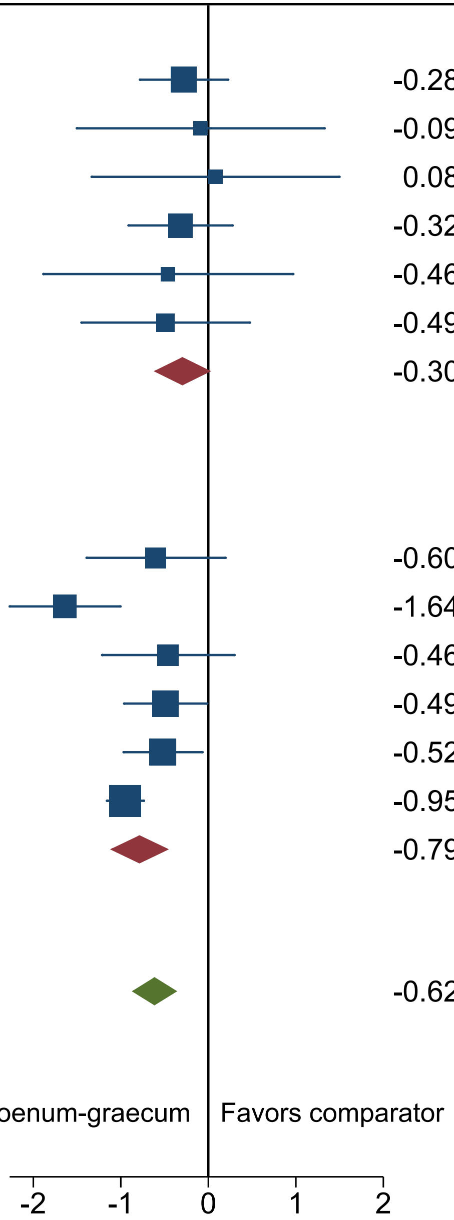

Random-effects REML model
